# Fatal Outcome of Widespread Cranial Basal Cell Carcinoma Due to Intraparenchymal Brain Extension in a Setting of Local Recurrences

**DOI:** 10.7759/cureus.112885

**Published:** 2026-07-17

**Authors:** Tayler Gant, Serguei Bannykh, David Frishberg

**Affiliations:** 1 Department of Pathology & Laboratory Medicine, Cedars-Sinai Medical Center, Los Angeles, USA

**Keywords:** basal cell carcinoma diagnosis, basal cell carcinoma of scalp, invasive basal cell carcinoma, morphea basal cell carcinoma, surgical excision of basal cell carcinoma

## Abstract

Basal cell carcinoma (BCC) is one of the most commonly encountered carcinomas; however, progression to invasive or locally advanced disease is extremely rare. The clinical course of locally advanced BCC is typically aggressive and has a poorer prognosis with variable survival. We present the case of a 58-year-old unhoused female with recurrent BCC of the left cranial vertex who underwent excision at an outside hospital. Intraoperative frozen section margins were negative for BCC; however, permanent sections demonstrated widespread residual tumor with morpheaform histology involving the parietal bone and aponeurosis at the deep margins. She subsequently underwent re-excision, multiple reconstructive procedures with grafts from the bilateral thighs, left forearm, and back, and immunotherapy with vismodegib. In September 2025, she presented with right upper extremity weakness and headache. Magnetic resonance imaging (MRI) revealed osteomyelitis and a left intra- and periventricular parietal fluid collection consistent with an abscess. She underwent left hemicraniectomy with wound debridement and full-thickness skin grafting. Histology demonstrated infiltrative nests and irregular strands of basaloid cells with peripheral palisading invading the skin, parietal bone, and brain parenchyma. Management included repeated wound debridement, full-thickness skin grafting, and consideration of adjuvant radiation to reduce recurrence after grafting. Three free-flap reconstructions with revisions were required because of infection, wound progression, and dehiscence. Persistent infection and a nonhealing wound ultimately precluded further surgical intervention. She was transitioned to comfort care and died in October 2025. This is a unique case of recurrent invasive and locally advanced BCC that supports the notion that this is an aggressive form of the more common indolent cancer seen in daily practice. Emphasis on early detection and intervention is vital in these particularly aggressive cases.

## Introduction

Basal cell carcinoma (BCC) is the most common malignancy and accounts for the majority of nonmelanoma skin cancers worldwide [1]. Most BCCs exhibit indolent growth patterns and have low metastatic potential; however, a subset (~1%-2%) can exhibit more aggressive behavior, presenting with local tissue destruction and invasion or, more rarely, distant metastasis [2]. Aggressive cases tend to have delayed presentation or recur after incomplete excision. Extremely locally aggressive or even metastatic growth may occur and can be treatment-resistant or unresectable [3]. Combination therapies are employed to control aggressive disease, including surgery, radiation, systemic targeted therapy, and immunotherapy [4,5].

Theragnosis of aggressive and advanced BCC is generally poor but depends heavily on the site of involvement and extent of growth. For instance, metastatic BCC most commonly arises in the head and neck region, with a tendency to have a large tumor burden [6]. Regional or nodal disease versus distant metastases has also shown significant differences in prognosis. This is partly due to location and dissemination patterns, specifically when the spread involves vital structures [7]. The recognition of these particularly high-risk clinical and histopathologic features is crucial for early diagnosis and intervention. As exemplified in our case, delayed presentation can adversely impact the outcome of aggressive and extensive BCC.

## Case presentation

**Figure 1:**
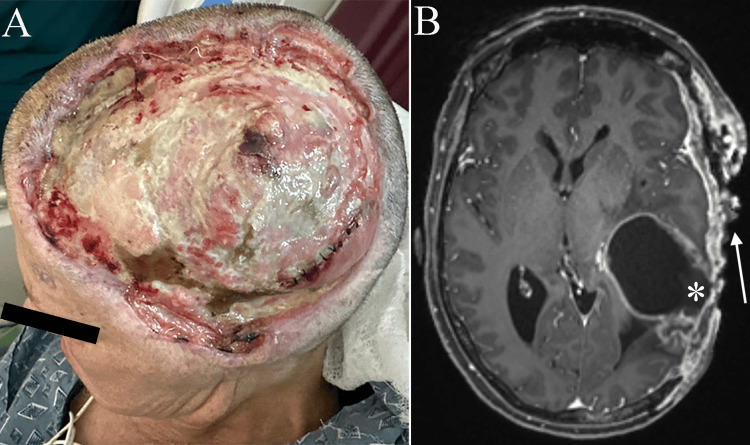
Basal cell carcinoma of the scalp with abscess (A) Pre-operative appearance of a necrotic skin defect over the left hemicranium. (B) MRI Axial T1 post-contrast gadolinium enhanced image showing skin and bone destruction with defect (arrow), dural and subdural enhancement, and left intraventricular fluid collection consistent with abscess (*).

**Figure 2:**
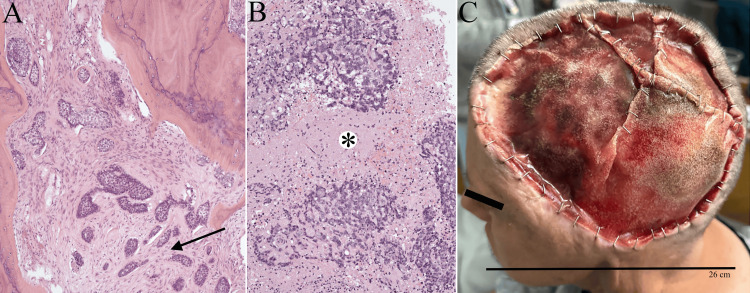
Invasive basal cell carcinoma of bone and brain (A) Basal cell carcinoma invading the bone, with morpheaform features (arrow); H&E, 100X. (B) Basal cell carcinoma invading the brain parenchyma (*); H&E, 200X. (C) Left cranial vertex graft.

A 58-year-old unhoused female with a history of recurrent BCC of the left cranial vertex (Figure 1A) underwent excision at an outside hospital, where she was originally diagnosed in 2016. Intraoperative frozen section margins were negative for BCC; however, subsequent additional sampling for permanent section analysis showed widespread involvement by BCC. The tumor had morpheaform histologic features, characterized by small clusters or nests of basaloid tumor cells infiltrating the dense, fibrotic stroma, with involvement of the parietal bone and aponeurosis at the deep margins. She subsequently underwent excision and multiple rounds of reconstruction with grafts from the bilateral thighs, left forearm, and back (Figure 2C), as well as immunotherapy with vismodegib.

In September 2025, she presented to our hospital with right upper extremity weakness and headache. Magnetic resonance imaging (MRI) revealed osteomyelitis and a left intra- and periventricular parietal fluid collection consistent with an abscess (Figure 1B). She underwent left hemicraniectomy with wound debridement and a full-thickness skin graft measuring over 26 cm in greatest diameter. Histology demonstrated nests of basaloid cells with peripheral palisading, characterized by tumor cells aligning along the outer edges of the nests and infiltrating the stroma in irregular strands. In addition to the skin, the tumor invaded the parietal bone and brain parenchyma (Figure 2A, B). Based on these pathologic findings, the tumor was staged as pT4N0M0. 

Wound cultures were positive for methicillin-resistant *Staphylococcus aureus *(MRSA) and *Proteus *species. Her treatment consisted of multiple wound debridements, a full-thickness skin graft (Figure 2C), and consideration of adjuvant radiation to control cancer recurrence after grafting. A total of three free-flap reconstructions with revisions were required because of infection, wound progression, and dehiscence. Persistent infection and her nonhealing wound precluded further surgical management. She was transitioned to comfort care and passed away in October 2025. 

## Discussion

BCC is a commonly encountered carcinoma associated with sunlight exposure and typically exhibits indolent behavior with low metastatic potential [1]. However, 1%-2% of patients will develop advanced disease due to delayed presentation or aggressive tumors that are resistant to treatment [2]. These tumors demonstrate local invasiveness and the potential to destroy soft tissue, cartilage, muscle, and bone. Critical factors associated with aggressive behavior include delayed presentation, recurrent disease, large tumor burden, high-risk anatomic location, and specific histologic subtypes [1].

Locally advanced BCC can be defined as a disease that is not eligible for surgery or radiation because of its degree of local invasion or recurrent disease following standard or first-line treatment [4,5]. Recent therapeutic advances and emerging therapies have changed management strategies, such as Hedgehog pathway inhibitors (HHIs), including vismodegib and sonidegib. Both are indicated as first-line treatment for locally advanced BCC, while vismodegib is also indicated for metastatic BCC [5]. Checkpoint inhibitors, particularly cemiplimab, have also played a role for patients whose disease progresses while on HHIs or who are intolerant of HHIs [5]. These advanced cases often require multidisciplinary management to optimize functional and oncologic outcomes, with collaboration among dermatology, surgical oncology, and radiation oncology.

The specific histopathologic patterns associated with a more aggressive nature or higher rates of recurrence are infiltrative, micronodular, morpheaform, and basosquamous subtypes [1]. Aggressive subtypes exhibiting greater subclinical extension have been identified during Mohs micrographic surgery, often requiring wider margins for clearance, specifically the infiltrative pattern of BCC [8]. Distinguishing histologic subtypes provides insight into cases in which locally invasive lesions may extend beyond clinically visible margins, leading to incomplete resections and recurrence. Another predictor of local invasion, proposed by Fernandez-Figueras et al., other than the traditional WHO histologic subtyping, is tumor budding. To assess for tumor budding, a threshold of ≥3 foci and ≤3 cells across all tumor sections is used [9]. This has been suggested to be a superior additional parameter for gauging biologic aggressiveness in locally advanced BCC [9]. In our case, the histology demonstrated morpheaform-pattern BCC, which coincides with one of the high-risk associated subtypes of BCC.

Imaging can also play a critical role in locally advanced BCC, specifically in the head and neck region, where invasion of sensitive structures is suspected. Recommendations are made by the American College of Radiology via the Appropriateness Criteria, which assess and determine imaging guidelines for specific clinical conditions [10]. For aggressive nonmelanomatous skin cancers with concern for deep structural invasion or bone involvement, CT or MRI is recommended [10]. Utilizing imaging modalities can facilitate accurate staging and surgical planning in cases with extensive tumor burden, such as in our case, which presented with brain and parietal bone invasion.

## Conclusions

Although BCC is commonly encountered and often associated with indolent behavior, a subset of carcinomas demonstrates aggressive local invasion and high morbidity. Locally advanced and infiltrative BCCs can be challenging because of local and distant extension, recurrence risk, treatment resistance, and involvement of important anatomical structures. Recognition of these high-risk features is essential for timely intervention and appropriate treatment management.

Recent advances in systemic therapies have expanded treatment options for patients with aggressive recurrent tumors or in cases that have been deemed unresectable. Surgical excision, with the goal of obtaining negative margins, remains the standard of management whenever possible. Brain invasion, lack of negative margins, severe and recurrent infection, and housing instability interfering with continuity of care contributed to disease progression and the subsequential outcome in this case. This case highlights the importance of early recognition, appropriate intervention, and adequate follow-up in preventing the progression of aggressive and locally destructive BCC.
